# Fatal Effect of Eau Medicinale

**Published:** 1811-03

**Authors:** John Ring

**Affiliations:** New Street, Hanover-Square


*To the Editors of the Medical and Physical Journal.*

---

*Fatal Effect of Eau Medicinale. Communicated by* Mr. Ring, Member of the Royal College of Surgeons in London.

Gentlemen,

The Eau Medicinale has acquired a high reputation for the cure of the gout; but the following case, in which it appears to have proved fatal, shews the necessity of a little more caution in the use of that medicine, than has hitherto been observed.

I am, Gentlemen,

Yours, &c.

JOHN RING.

*New Street, Hanover Square,*
*Jan.* 31, 1811.

---

Mr. Smith, of Bishop's-yard, Charles-street, Grosvenor-square, sixty-five years of age, was troubled with the gout when twelve years old; and in general had it twice a year, for the last twenty-five years. He frequently had it in his stomach as well as in his limbs.

In the spring of 1810, he took half a bottle of the Eau Medicinale, which operated as a cathartic; and in the space of twelve hours, he was much relieved.

On Sunday, the 25th of November, he was seized with the gout in his foot; but was not confined to his bed. On Wednesday, the 28th, he took half a bottle of the Eau Medicinale, which operated violently as an emetic, a cathartic, and a sudorific. On Thursday his foot was well; but he complained of a pain in the pit of his stomach, which he could cover with his finger. This increased till five o'clock, when it became very violent, and continued so during the night; alternately affecting his stomach and his bowels.

On Friday the pain gradually abated; on Friday night the black vomiting came on, and on Saturday afternoon he expired.

The medicine, which seems to have proved fatal in this case, was bought at Mr. Befort's, in St. James's-street; and there is no reason to doubt that it was genuine. The mischief seems to have been occasioned by the patient's taking a larger dose than his constitution could bear.

The printed paper accompanying the Eau Medicinale, directs laudanum to be taken when the operation is too violent; but this he neglected.

